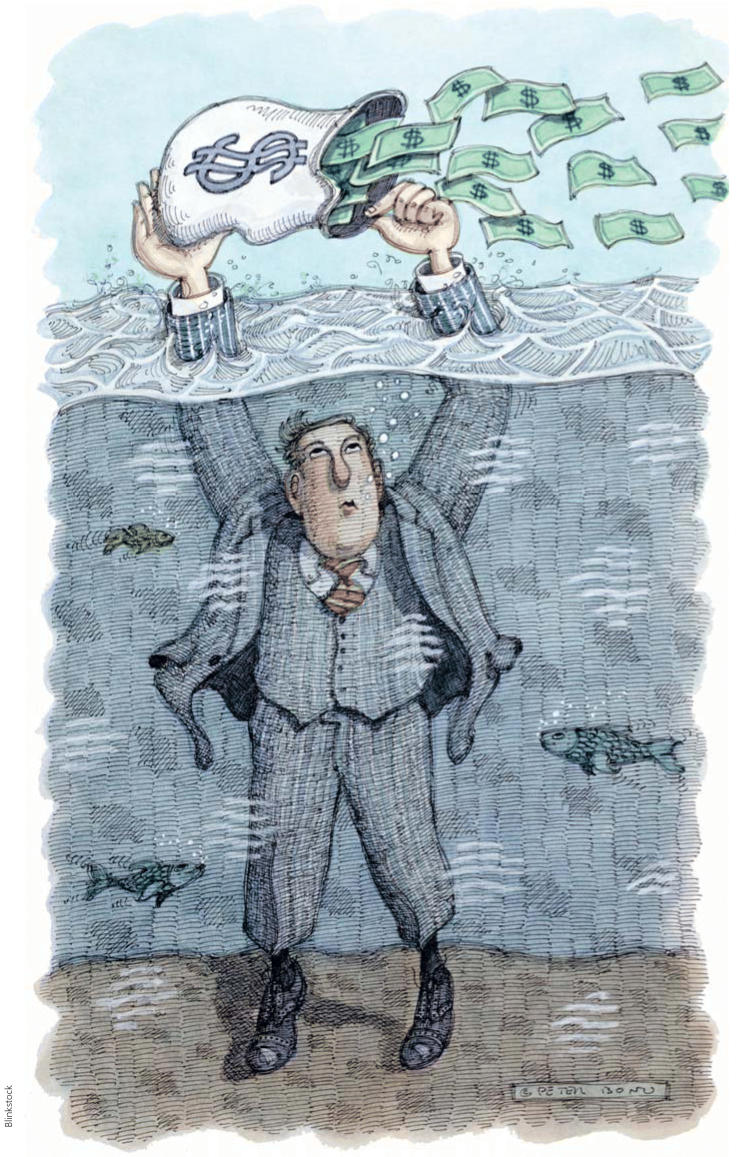# A Risky Environment for Investment

**DOI:** 10.1289/ehp.114-a478

**Published:** 2006-08

**Authors:** Dinesh C. Sharma

Floods in Europe. Heat waves in the United States. Snowfall in the deserts of the United Arab Emirates. These are among the unusual weather conditions witnessed in different parts of the world in the past five years, conditions that demonstrate how climate change is beginning to impact people. While governments negotiate targets for cutting down emissions of greenhouse gases—seen by bodies such as the Intergovernmental Panel on Climate Change as the most viable mitigation measure to slow down the processes causing global warming—the fallout from rapid climate change has already set alarm bells ringing in the financial sector.

Institutional investors are realizing that taking environmental, social, and corporate governance, or ESG, issues onboard is in the long-term interest of the investments they hold. Not doing so could pose a financial risk to their investments.

Yet, in the absence of any pressure from market regulators to disclose information on environmental issues, and given the focus of markets on short-term profit, companies are not always forthcoming with full disclosures on environmental risks. According to a May 2006 report titled *Climate Risk and Energy in the Auto Sector: Guidance for Investors and Analysts on Key Off-Balance Sheet Drivers*, by the Ceres network for socially responsible investment (SRI), investors and analysts are finding it difficult to assess automotive companies due to lack of disclosure from companies and uncertainty about the future course of U.S. energy and climate change policies.

At the same time, market research firms—which give investors “buy” and “sell” advice—need to be educated about climate change and other nonfinancial risks. In the February 2004 study *Values for Money: Reviewing the Quality of SRI Research*, the European action groups SustainAbility and Swedish Foundation for Strategic Environmental Research showed that only 3 of 35 stock market research firms specializing in SRI actually analyzed the link between ESG issues and material impacts on investment value drivers. Most used generic research methodologies and gathered data primarily from the companies themselves with little, if any, verification.

Today a number of initiatives seek to weave ESG factors into virtually every segment of the market. Most recently, the UN launched the Principles for Responsible Investment (PRI), and a pact for financial institutions known as the Equator Principles was just revised to broaden its scope and thereby extend environmental protection. The blending of sustainability and profitability can, however, seem at times an uneasy marriage, at others a battle royale.

## The Economics of Disaster

The frequency of floods, droughts, severe heat waves, and violent windstorms has increased significantly in the last decade. Between 1998 and 2004, Europe suffered more than 100 major damaging floods that killed 700 people, displaced half a million others, and caused more than US$31 billion in insured economic losses, according to the European Commission. The European Environment Agency’s 2004 report *Impacts of Europe’s Changing Climate* pointed out that climate change is likely one of the causes of flooding in Europe.

The losses due to natural calamities, many of them related to climate change, grew to $46 billion per year in the 1990s, up from $4 billion per year in the 1950s. By 2004, the figure had more than doubled to $107 billion, then spiked to $123 billion in 2005, mainly due to Hurricanes Katrina and Rita, according to *Climate Change Futures: Health, Ecological and Economic Dimensions*, a November 2005 report prepared by the Center for Health and the Global Environment at Harvard Medical School. Resurgence of infectious diseases such as malaria and dengue, shortage of drinking water, and reduced agricultural production due to outbreaks of pests and diseases are among long-term impacts of climate change pointed out in the report.

Insurance companies are beginning to look at climate change as a long-term risk, while banks are revising their lending guidelines to align them with risks related to climate change. The insurance industry could play a key role in devising mitigation strategies. “[I]nsurers founded the early fire departments and owned the equipment . . . , helped establish the first building codes and stand behind consumer-safety organizations such as Underwriters Laboratories. Loss prevention is ‘in the DNA’ of the insurance industry,” observed the authors of the Harvard report.

Big corporations in sectors like electric power and the automotive industry are under greater scrutiny from bankers, shareholders, and action groups with regards to their strategies to cut greenhouse gas emissions and other environmental risks. They are under pressure to disclose enough information on these matters so that investors can take into account risks to their portfolios.

In the first half of 2006, about 180 ESG-related shareholder resolutions were either listed or presented in corporate meetings in the United States, according to data collected by the Social Investment Forum, an SRI trade body. Of 32 resolutions that related to global warming issues, 12 were withdrawn after the receiving firms committed to produce or disclose the requested information.

“All these actions are significant because they have influenced the companies to review the issue more closely and to report more fully to shareholders and the public. That is the necessary first step for companies to understand and reduce their climate change risks,” says Meg Voorhes, director of social issues services at Institutional Shareholder Services, a firm providing proxy voting services.

Climate change is not the only environment-related financial risk that active shareholders are concerned about. At the annual shareholders meeting of Dow Chemical Company on 11 May 2006, a group of investors forced voting on a resolution that asked Dow to take steps to address ongoing environmental and health problems relating to the 1984 Bhopal gas disaster. The investors—which included the New York City Fire Department Pension Fund, the New York State Common Retirement Fund, and Boston Common Asset Management—feared that if Dow did not take any action, it could be risky for its reputation and business in India and Asia. The resolution received 6.3% of the vote—not enough to pass, but enough to ensure it is presented again next year.

“The longer Dow Chemical fails to address the lingering human issues related to the Bhopal tragedy, the greater the potential negative impact to its long-term profitability,” observed Alan G. Hevesi, sole trustee of the New York State Common Retirement Fund, in a press release issued by Amnesty International USA. “As a fiduciary, I am concerned that if Dow does not put this problem to rest, it could hurt the company’s current and future business relationships in India’s huge and rapidly expanding market and around the world.”

## Creating New Tools for Investors

Since the 1992 UN Conference on Environment and Development, the UN has been working with businesses and industries to make their activities environmentally sustainable. A number of international treaties and agreements are under implementation or negotiation. At the same time, the UN Environment Programme (UNEP) also began working with banking and financial sectors to help them integrate environmental considerations into their operations and services as well as to boost investment in eco-friendly technologies. In 1995, a similar drive was launched for the insurance sector.

All these actions are significant because they have influenced the companies to review the issue more closely and to report more fully to shareholders and the public. That is the necessary first step for companies to understand and reduce their climate change risks.

–Meg Voorhes, Institutional Shareholder Services

Since 2003, the banking/finance and insurance programs have operated under a common umbrella—the UNEP Finance Initiative (FI). This initiative is based on the belief, outlined in its mission statement, that “sustainable development is best achieved by allowing markets to work within an appropriate framework of cost-efficient regulations and economic instruments.” At present, the UNEP FI has more than 230 signatory institutions from 45 countries. [For more information on the UNEP FI, see “EHPnet: UNEP Finance Initiative,” p. A465 this issue.]

Participants in a separate UN program called the Global Compact have developed a set of 10 principles in areas such as human rights, labor, environment, and anticorruption. Whereas the UNEP FI concentrates on financial institutions, the Global Compact, begun in 2000, works with industry and business directly. The Global Compact acts as a body to promote corporate social responsibility based on common principles for businesses.

There have been other, non-UN initiatives as well, like the Equator Principles. Ten leading banks from seven countries first adopted the Equator Principles in June 2003. These principles are a set of guidelines developed by the banks for managing ESG issues related to the financing of development projects with capital costs of US$50 million or more (this cap was reduced to US$10 million on 6 July 2006). Under the principles, investment projects are vetted using a process based on the environmental and social screening process of the International Finance Corporation.

“An evaluation of financial sector engagement shows a significant shift in the way financial institutions view these issues,” observes Paul Clements-Hunt, head of unit for the UNEP FI. “They have moved from a largely public relations focus of the early 1990s to the commencement of mainstreaming of sustainability and social responsibility issues in their core business lines.”

The UN’s PRI, launched on 27 April 2006, represents one of the latest efforts to integrate sustainability and profitability. The PRI are specifically intended for pension funds and large institutional investors. So far about 50 U.S. and European asset owners and fund managers representing funds to the tune of US$4 trillion have signed on to the PRI. Pension funds from developing countries will also be encouraged to sign up in the future.

Public and private pension funds constitute an important segment of financial markets, accounting for up to 35% of total global investment. The PRI stemmed from the recognition that while investors are becoming aware of risks posed to their investments due to ESG issues, they do not have a framework or common guidelines to work on these issues with the companies they are investing in. Also, companies that take proactive measures on these issues are insufficiently rewarded by markets, which continue to be driven by short-term considerations.

These newest principles—which were developed by an international group of more than 20 leading pension funds, foundations, and special government funds—are an attempt to correct this disconnect. “They provide a framework for achieving better long-term investment returns and more sustainable markets. If implemented, they have tremendous potential to more closely align investment practices with the goals of the UN,” noted UN secretary general Kofi Annan at the launch of the PRI at the New York Stock Exchange.

## Behind the PRI

The PRI were founded on the premise that institutional investors have a duty to act in the best long-term interests of their beneficiaries. As Clements-Hunt puts it, “PRI provides the thinking and guidance, while individual funds provide the meat on the bone in terms of their own national or regional context.”

Jon Sohn, a senior associate at the World Resources Institute, elaborates upon this role: “What PRI does at its core is send a top-down signal to asset managers of funds to integrate these issues into how they pick stocks and analyze companies. . . . This is an indirect way to influence companies, as it impacts valuation decisions, which in turn impacts what companies think is important to investors. The key challenge is demonstrating the ‘materiality’ of sustainability issues and linking that to all the money behind these investors. The potential is great.”

Under the PRI, institutional investors would incorporate ESG issues into their investment analysis and decision-making processes as well as into ownership policies and practices of institutional investors. Investors would seek appropriate disclosure on ESG issues by the entities in which they are investing. They would also promote the principles within the investment industry and monitor progress in their implementation. Finally, they would work together to enhance the effectiveness of implementing the principles.

The principles suggest 35 possible actions that institutional investors and asset managers can take to integrate ESG considerations into their investment activities. These include requesting that investment service providers (such as financial analysts and brokers) integrate ESG factors into evolving research and analysis; developing an active ownership policy consistent with the PRI and exercising voting rights or monitoring compliance with voting policy; asking investment managers to work with companies on ESG-related issues; asking entities in which institutional investors invest for standardized reporting on ESG issues; and requesting information from companies regarding adoption of and adherence to relevant norms, standards, codes of conduct, or international initiatives. Signatories to the PRI are required to report on implementation or provide an explanation if they do not comply with the principles.

The PRI Investor Group, the UNformed body that developed the principles, is now working on a set of specific short-and intermediate-term tools to support their interpretation and implementation. These are likely to include means of assessing and comparing the extent to which fund managers are dealing with ESG issues in their investment processes and contact with companies; an online resource for signatories, with advice on different means of implementing the PRI for different asset classes and investment styles; and a platform for collaborative engagement with companies in which signatories jointly invest.

The principles are voluntary and thus represent a self-reporting system. “The voluntary nature was necessary in order to achieve consensus in a sufficiently large group. We considered voluntary guidelines to be more flexible and thus better able to adapt to changing circumstances,” explains Colin Melvin, chairman of the PRI Investor Group. “The higher standard of definitional clarity required by mandatory guidelines would have been impractical to achieve in the time frame available to us, given the very many asset classes and investment styles represented by the signatories’ activities.”

But translating commitments into action will require significant policy changes at the investor level. In May 2006, South Africa’s Government Employee Pension Fund announced new measures following its adoption of the PRI. Fund chairman Martin Kuscus says the fund will now promote increased investor activism and elect independent directors to the boards of companies in which it holds significant investments. It will also monitor and rate companies for their performance on ESG issues, according to a 26 May 2006 report in the business magazine *Personal Finance*.

Other experts and action groups feel that the principles need to be accompanied by policy changes at the national level in order to be effective. “Some of the major electric generating companies have come out in favor of policies to control emissions. But it is clearly hard for them to take steps to reduce emissions voluntarily if it makes them less competitive in the marketplace,” says Ashok Gupta, air and energy program director at the Natural Resources Defense Council. “These principles are helpful in moving the market in right direction, but they are not a substitute for meaningful government policies.” He adds, however, that some companies are voluntarily pursuing measures to make themselves more efficient and reduce their costs.

## What’s Next?

Right now investors are focused on disclosures—getting information from companies on environmental issues. But making a difference on the ground demands going beyond disclosures and investing in cleaner technologies and sustainable growth. Often pressure from civil society and consumer groups can bring faster results.

For example, in April 2006 Greenpeace reported on its investigation into how Amazon rainforests are being cleared up to make way for production of soybeans, meant for use as feed for chickens and pigs in Europe. These animals become fast food products sold by McDonalds, KFC, and other restaurant chains. Greenpeace alleged that the International Finance Corporation wrongly assessed a loan to Grupo Andre Maggi, which controls major soybean production in Brazil, as being of “low environmental risk.” And based on this assessment, Rabobank lent more than US$330 million to the Brazilian company.

Responding to the criticism, McDonalds has assured corrective action. “We are very committed to purchasing practices that do not impact the valuable Amazon biome. We have a strict policy regarding this in beef. New developments have shown possible linkages to soya production affecting the Amazon, so we are now working with our suppliers so that if this is the case, our supply of soya ingredients will not come from such areas,” says Bob Langert, senior director of corporate social responsibility at the company.

An evaluation of financial sector engagement shows a significant shift in the way financial institutions view these issues. They have moved from a largely public relations focus of the early 1990s to the commencement of mainstreaming of sustainability and social responsibility issues in their core business lines.

–Paul Clements-Hunt, UNEP Finance Initiative

Still, some green groups doubt that programs such as the PRI will actually achieve very much. In the 28 April 2006 edition of the British newspaper *The Guardian*, Friends of the Earth corporate campaigner Craig Bennett said, “It seems we get some kind of funky new initiative every other week. Voluntary initiatives make very little difference, if at all. Do we really think that in the boardroom when it comes to crunch decisions about competitiveness, lowering costs, and sourcing, that they have any impact?”

This response may reflect these groups’ experience with other programs launched earlier. ABN AMRO—the global banking group that led the implementation of the Equator Principles—and the European Bank for Reconstruction and Development are in the thick of a controversy over their proposed lending for the Sakhalin II oil and gas project in the Russian Far East. If the Equator Principles allow lending to a project with an environmental risk as significant as the potential disappearance of an entire whale species, as is the case of Sakhalin II, the very relevance of such principles is at stake, pointed out *Principles, Profits, or Just PR?*, an April 2006 report by Banktrack, a Netherlands-based network of NGOs that tracks the impact of private finance. In the end, some groups feel that divestment is a better strategy than engaging with a company and trying to change its environmentally damaging ways.

The marriage of profitability and sustainability has only just begun. “[T]he logic of responsible investment—i.e., the deliberate incorporation of material social and environmental considerations in investment decision-making—has yet to be embraced by the wider investment community,” noted the authors of *Mainstreaming Responsible Investment*, a study commissioned by the World Economic Forum in 2005. “Responsible investing remains a boutique segment of the industry despite widespread, if largely anecdotal, evidence that social and environmental factors affect market valuations both positively and negatively.”

Attention to nonfinancial factors within the wider investment community remains largely reactive and episodic. Changing this to put social and environmental concerns in the forefront remains a daunting challenge. The multitrillion-dollar question is: can profitability and sustainability coexist? The answer from the PRI signatories is: they must.

## Figures and Tables

**Figure:**